# Quinine Prophylactic of Puerperal Fever

**Published:** 1848-11

**Authors:** 


*Quinine prophylactic of Puerperal Fever.*—This idea that quinine is a preservative against puerperal fever was started by M. Alphonse Leroy, of Rouen, in 1793. M. Leudit put it to the test in an epidemic which occurred in 1843, and lasted for three months, administering it before the accustomed period of the first appearance of the malady. For this purpose he employed the quinine in 15 grain (one gramme) doses, and in the few cases it was then tried in, no fever followed. He repeated his experiments in two other epidemics, occurring in the years 1845 and 1846, when he found that those who submitted to this medicine did not contract the fever. To give the statistics :—Of 83 women who entered the Hotel Dieu de Rouen, between September, 1843, and January, 1844, 74 took no medicine, and 21 of them were seized with puerperal fever, while the remaining 9 were dosed with the quinine, and escaped contagion. Again ; between July 8th and August 9th, 1845, 26 deliveries occurred : 11 women were submitted to no medication, and eight of them were attacked with the epidemic fever ; of the 15 others, treated with sulphate of quinine, one only caught the disease. Lastly, between the 9th of March and the 21st of April, 1846, 36 women were delivered : of the 19 who took no quinine, 11 were attacked ; of the 16 submitted to its action, only one was seized with fever.

The following is the manner in which M. Leudet employs the quinine : as soon as the newly delivered woman has a little recovered the shock of child-birth—viz., in about four hours after delivery, 15 grains of the medicine are given in the course of the 24 hours, in three portions. The same quantity is prescribed the next day, but on the third day it is diminished to ten grains, and the same dose is persevered in until the usual period of the accession of the fever has passed by, up to about the sixth day. The occurrence of milk fever is not always an indication to stay the quinine, for in very many cases that febrile disturbance is very slight.

The plan of using quinine as a prophylactic has been subsequently adopted in Paris by M. Cazeaux, who could, from his experience, however, make no report of its efficacy. Nevertheless, any remedy holding out such a promise, in so fearful a disease, should not be thrown aside until after a careful and repeated trial. On the other hand, hygienic measures must be looked upon as by far the best safeguards, both against the developement and the propagation of the puerperal fever.—*Lon. Lan.*

